# *‘It’s what you do that makes a difference’* An interpretative phenomenological analysis of health care professionals and home care workers experiences of nutritional care for people living with dementia at home

**DOI:** 10.1186/s12877-019-1270-4

**Published:** 2019-09-10

**Authors:** Louise Mole, Bridie Kent, Mary Hickson, Rebecca Abbott

**Affiliations:** 1 Institute of Health and Community, School of Health Professions, University of Plymouth, Plymouth, UK; 2 Collaboration for Leadership in Applied Health Research and Care, South West Peninsula (PenCLAHRC), The National Institute for Health Research (NIHR), Plymouth, UK; 3 School of Nursing and Midwifery, University of Plymouth, Plymouth, UK; 4 Centre for Health and Social Care Innovation, University of Plymouth: an affiliated centre of the Joanna Briggs Institute, Plymouth, UK

**Keywords:** Dementia, Nutrition, Health care professionals, Interpretative phenomenological analysis, Home care

## Abstract

**Background:**

People living with dementia at home are a group who are at increased risk of malnutrition. Health care professionals and home care workers, are ideally placed to support nutritional care in this vulnerable group. Yet, few, if any studies, have captured the experiences of these workers in respect of treating and managing nutritional issues. This interpretative phenomenological study aimed to explore the experiences and perceptions of the nutritional care of people living with dementia at home from the perspectives of health care professionals and home care workers.

**Methods:**

Semi-structured interviews were conducted between December 2017 and March 2018, and supplemented with the use of a vignette outlining a scenario of a husband caring for his wife with dementia. Health care professionals and home care workers were purposively recruited from local care providers in the south west of England, who had experience of working with people with dementia. An Interpretative Phenomenological Analysis (IPA) approach was used throughout.

**Results:**

Seven participants took part including two home care workers, a general practitioner, dietitian, occupational therapist, nurse and social worker. The time in their professions ranged from 3 to 15 years (mean = 8.9 years). Following analysis, four superordinate themes were identified: ‘responsibility to care’, ‘practice restrained by policy’, ‘in it together’, and ‘improving nutritional care’. This group of health care professionals and home care workers recognised the importance of improving nutritional care for people living with dementia at home, and felt a responsibility for it. However they felt that they were restricted by time and/or knowledge. The importance of supporting the family carer and working collaboratively was highlighted.

**Conclusions:**

Health care professionals and home care workers require further training to better equip them to provide nutritional care for people living with dementia at home. Models of care may also need to be adapted to enable a more flexible and tailored approach to incorporate nutritional care. Future work in this area should focus on how health care professionals and home care workers can be better equipped to screen for malnutrition, and support changes to nutritional intake to mitigate malnutrition risk.

**Electronic supplementary material:**

The online version of this article (10.1186/s12877-019-1270-4) contains supplementary material, which is available to authorized users.

## Background

There are an estimated 50 million people living with dementia globally [1]. For those living with dementia at home, family carers take on the responsibility of managing health, emotional and social needs. These can become more complex and demanding as the dementia progresses, and can have profound impacts on the individual and their family [2].

For someone living with dementia, nutritional status can be affected in many ways including changes in memory, motor skills, taste, and appetite and swallow function [3, 4]. The presentation of symptoms will vary amongst individuals as the disease progresses, and may also be dependent on dementia type (e.g. Alzheimer’s disease or vascular dementia) [5]. The initial identification and management of nutritional issues are often reliant upon the family carer. This is one element of care amongst many others that an inexperienced carer may have to consider, increasing the risk of elevated levels of burden and stress, which may impact upon the quality of care provided, as well as emotional wellbeing [6].

Carer surveys have indicated that there is a need for increased primary care support relating to the nutritional needs and consequences associated with dementia in those living at home [7]. Family carers are conscious about ‘doing the right thing’ when it comes to providing nutritional care, however feel uncertain about the food choices they are making [8]. To reduce the ‘care-burden’ experienced by family caregivers, domiciliary home care support may be used, and can contribute to helping someone with dementia maintain an adequate nutritional status whilst living at home. Best practice guidelines have been published in the UK to support managers of home care agencies [9], however there is limited literature that evaluates the nutritional care that these agencies provide. There is also a paucity of studies examining the views and experiences of home care workers, and other health care professionals who may interact with people living with dementia at home (e.g., general practitioner’s (GP’s), community social workers, and community dietitians).

The reason for the delayed identification of malnutrition risk and the inconsistent monitoring of nutritional status could be because some health care professionals perceive a lack of benefit to the patient (i.e. taking a nihilistic view) [10]. However, the timely identification of nutritional issues, regular monitoring of nutritional status, and increased support and education for all types of carers and health care professionals involved with people living with dementia at home are important factors, highlighted in a recent review of the literature [11].

The aim of this study was to understand the experiences and perspectives relating to nutritional care of health care professionals and home care workers, who interact with people living with dementia at home. Interpretative phenomenological analysis (IPA) was chosen as the research methodology. IPA seeks to examine, as far as is possible, the perceptions of the participant. However, the process also involves the interpretative activity of the researcher, otherwise known as ‘double hermeneutic’. Therefore, in-depth interpretative accounts for a small number of participants are presented when using IPA, instead of a generalised account for a larger sample.

## Methods

### Study design

Each participant took part in one semi-structured interview between December 2017 and March 2018. As part of the interview, a vignette was used that had been specifically developed for this research. All participants were shown and read the same vignette, which outlined a fictitious scenario of a husband caring for his wife with dementia at home (see Additional file 1). The vignette had been piloted with a group of health care professionals prior to interviews, to ensure internal validity. Vignettes have been shown to be useful in eliciting awareness and attitudes in health care research, as they offer a level of depersonalisation allowing the interviewee to think beyond their own professional circumstances [12]. The consolidated criteria for reporting qualitative research (COREQ), was used during the design of this study [13].

### Participants

Following ethical approval from the University of Plymouth Faculty of Health and Human Sciences Research Ethics Committee (16/17–778), a variety of health care professionals and home care workers were purposively recruited from the lead author’s professional network.

To be eligible for inclusion participants were: health care professionals and home care workers residing in South-West England, who had experience engaging with people living with dementia at home. Written consent was provided prior to interview, and verbal consent was also audio recorded.

Seven health care professionals and home care workers volunteered to take part in the study with an average duration in their profession of 8.9 years, and were predominantly female (1 male, 6 female) (Table 1). This sample size aligns with other IPA studies involving health care professionals, to provide a manageable number of detailed individual accounts [14, 15]. This study did not intend to generate theory, therefore theoretical saturation (or data saturation) was not considered [16].

**Table 1:** Participant demographics

Profession	Code	Male/Female (M/F)	Time in profession (years)
General Practitioner	GP	M	15
Community Social Worker	SW	F	3
Community Occupational Therapist	OT	F	3
Community Dietitian	CD	F	15
Community Nurse	CN	F	11
Care Worker 1	CW1	F	8
Care Worker 2	CW2	F	7

### Data collection

Participants were interviewed at their own home, at their place of work (outside of working hours), or on university premises according to participant preference. Interviews lasted between 20 and 39 min, and were audio recorded and transcribed verbatim. An interview schedule was developed and piloted, and used for prompting where necessary (see Additional file 2). Topics included exploring the nature of their role, their experiences of nutritional care in this group, and what they felt would improve nutritional care for people living with dementia at home.

### Analysis

Data were analysed in accordance with an IPA methodology by LM using NVivo 11 [17] to aid coding and organise emergent themes. Each account was read and re-read, ensuring that any new ideas and insights were generated [18], and semantic content and language use were explored. Connections across themes were then identified before the next participant account was approached and the themes that emerged from the previous case were ‘bracketed’ [14]. Once all accounts had been analysed, patterns across accounts were investigated and superordinate themes created that captured the shared experiences of the participants. Throughout analysis, emergent and superordinate themes were discussed with MH, BK and RA. This systematic approach ensured traceability of the development of themes from participant’s original accounts.

### Authors’ perspectives

LM led the interviews, transcription and analysis and kept a reflective diary throughout the process. The research team have clinical and research experience in the field of dementia and nutrition, and LM is a registered dietitian. The impact of an existing professional relationship between the interviewer and interviewee was taken into account during data analysis through the researcher’s reflexive diary entries.

## Results

Four superordinate themes were identified that brought together the data from the seven participants (see Table 2). One theme (*responsibility to care*) was identified in all seven transcripts. However, three themes (*in it together [6/7 participants], practice restrained by policy [5/7]*, and *improving nutritional care [5/7])*, although clearly identified in some transcripts, were not evident in all. The vignette offered the opportunity for participants to explore their own feelings regarding a scenario which many had experienced in their professional practice. Some of this insight contributed towards the themes, however the response to the vignette is summarised separately.

**Table 2:** Superordinate themes with sub-themes

Responsibility to care	Practice restrained by policy	In it together	Improving nutritional care
• Care is a constant • Dealing with dementia as a GP • Monitoring and auditing • Promoting an active role • Responsibilities and measures • Role of the district nurse • The attributes you need to care for someone with dementia • The extended role of health professionals • The role of carers • The role of the social worker	• Time is limited • The value and disvalue of other support services • Tensions and frustrations • Resource Policy and Care Provision Models • Power removed • Assessments and Care Plans • A happy social environment can’t be measured	• Family carers need as much support • How care workers can support • Male carers cope better than females • Nutritional care is everyone’s responsibility • Togetherness • We are all in it together • Working with family caregivers • Worry and concerns involved with nutritional care	• Taking a problem-solving approach • Opportunities for improvement • Meal delivery is not enough • Malnutrition management • Making it better • Generational changes in nutritional knowledge • Awareness vs. knowledge

### Theme one: responsibility to care

This theme explores how the participants perceive the role that they play in supporting people with dementia living at home, and the responsibilities they feel they have to enable this. It examines the extended role that health care professionals and home care workers may have, and the attributes that participants believe are important for working with this population.

Although the specific role of each health professional differs, there was a commonality expressed by participants linked to ensuring a safe environment and promoting wellbeing. Often, this was assessed against an established framework or screening tool, such as the ‘Care Act’.

‘Our function is that we'd go out, assess the person against the Care Act, which has quite clear eligibility … Statutory-wise it would be safeguarding. We'd have a duty of care making sure that they're safe, well, and at home if home is the best environment for them.’ (SW)

All professionals felt a ‘responsibility to care’, and the GP in particular ardently expressed the magnitude of this responsibility.

‘Oh my goodness! I'm basically responsible for their whole care.’ (GP)

Some professionals perceived their role as not limited to their specialism, but recognised the importance of exercising an extended role, especially as they may be the only health care professional to have visited the person with dementia at home in a while. The community dietitian expressed how they would focus on nutrition, but also observe other health factors during a visit.

‘It’s nutrition but then also eyes. We're the eyes of - Somebody might not have seen a GP for a long time or have seen nobody for a long time. You're just looking around and making a judgment generally … For example, one of the questions I would routinely ask is what's your skin like? Are your pressure areas intact and if there's any concerns about that then it's a case of okay who is seeing that? Does anybody know about it?’ (CD)

Home care workers are amongst the professionals who may visit people at home on a regular basis, and describe their role as encouraging enablement and promoting an active role for the individual with dementia. This was particularly relevant when discussing food preparation, and one care worker described how important they felt it was to involve the person with dementia in this process. This was, however, caveated by the recurring issue of time constraints with delivering care.

‘I just think that with dementia, to get their minds working as well and actively doing something, I think cooking - maybe someone could go in and cook twice a week with them and make meals up that can go in the freezer … So getting them actively involved, you'll see a significant difference, you would, because their mind's - they're using their mind rather than letting it seize up.’ (CW1)

Some participants discussed the important attributes that they feel are required when working with someone with dementia. ‘Patience’ was cited as one of these attributes, and the community nurse described how this was important if visiting a patient around mealtimes.

‘We try not to do our visits around mealtimes, because they need to be protected really, especially with somebody with dementia that might take a long time to eat their meals. Some people even forget how to use a knife and fork, so you don't really want to be interrupting that … You need a lot of patience, got to have patience. If people haven't got patience, then you can't support people if you haven't got patience with dementia.’ (CN)

### Theme two: practice restrained by policy

This theme explores the impact that hierarchical influences, such as policies and care provision models, had upon the participants’ practice. It explores the issues expressed regarding time constraints concerned with providing adequate nutritional care, and how other support services can be helpful or a hindrance. The level of policy, models, assessments and care plans was an area of focus for many participants, and resulted in feelings of tension and frustration. Some expressed feeling a dissolution of power, where senior management made all decisions regarding care provision. The inability to effectively measure important outcomes, such as ensuring a happy, and social environment, was seen as a limitation of the health care system, which was perceived as being focused only on measurable outcomes.

Issues with time constraints was mentioned by a few participants, however the home care workers were most sensitive to how time can impact upon their role in the context of nutritional care for the individual with dementia.

‘As a carer, I know that sometimes you only have a certain amount of time to go in. A lunch call could be half an hour. In that time with someone with dementia you might have given them so many options and they choose one and then they decide they don't want that... It's just unfortunate that support workers are only given a certain amount of time and the people that dish out the time will be sitting there saying well hold on a minute, she's got dementia, she's got carers three times a day. That's where ours stops. Well it shouldn't. That's not a duty of care. Duty of care is to promote independent living at home.’ (CW1)

Dementia was expressed by some participants as a ‘social care issue’, and one which requires multidisciplinary input. Memory café’s (which support people with dementia and their family members in a safe, social setting) were viewed as a valuable resource by the GP, and one which differs to that of formal health provision services, such as memory clinics.

‘Memory cafes are very popular because they're not them and us, type of thing. It's very - they're very level playing fields. Whereas, when you go to the memory clinic, the consultant, the specialist, he's them and we're the patient.’ (GP)

Many participants described their roles framed within referral policies and procedures. This was deemed necessary to manage a large number of referrals with limited resources, but there were challenges associated with this, particularly in the context of changing strategies to prevent hospital admissions.

‘We would put an individualised nutritional care plan in place and then we would monitor that. We aren't able to monitor as closely as we would like because of the level of the dietetic service … I'm a bit biased, but I think we need more dietitians.’ (CD)

The tension between the cost of nutritional care provision and client requirements was perceived as an issue in social care. Many clients will be supported by meal delivery services. Although a cost-effective option, social workers are aware that this does not meet many of their client’s needs, who would perhaps benefit from increased carer support during mealtimes.

‘It's really quite hard, because of the budgets and stuff like that, they want to cut down on care. So, when we put care packages in, it has to be timed. They don't like us putting in carers to cook a meal. So, instead, they will say Community Meals or microwave meals.’ (SW)

Measured outcomes and targets are not reflective of the benefits of social interaction, as perceived by one of the home care workers. They serve a purpose, however the importance of creating an environment where people are able to interact to promote improved wellbeing, may produce positive yet unmeasurable outcomes. This insight does not align with a time-based model of care provision.

‘I don’t think we can ever underestimate social interaction. I think there is so much now based on outcomes, but we don’t actually appreciate enough just socially how important that is … we have targets and of course we can always have things to work to, but I think the fact that we can provide an environment to come in and socialise and be happy, because obviously all those things affect your mental health, your wellbeing.’ (CW2)

### Theme three: in it together

This theme explores how participants recognise the importance of the partnership that is required between themselves and family carers of people living with dementia at home. It describes how some family carers may cope better than others, and how participants feel they provide help and support with regards to nutritional care. Nutritional care is perceived by many participants as being a collective responsibility, in which everyone can play a part and it is recognised how health care professionals, home care workers and family carers need to work together.

The recognition of how family carers cope with providing nutritional care for someone living with dementia at home was noted by a home care worker in response to the vignette. The requirement for there to be as much support for the family carer as for the person with dementia was also noted.

‘I think that must be the hardest bit is the guilt, that they have the mixed emotions of what they must go through. They must go through so much frustration. They must get to the point where they get so exasperated by it all. That would be perfectly what you'd expect. I think he'd need just as much support in a different way.’ (CW2)

Male family carers were perceived by one participant to cope better than females. The occupational therapist who perceived this suggested this was due to females having carried out a ‘caring’ role for the family throughout their lives, and may feel more of a burden as a result of caring for a male spouse with dementia. Male family carers may take to unfamiliar tasks such as cooking and shopping with an organised and methodical approach.

‘Just the change in the role is a really difficult thing for a carer to take on, but I personally always think that men manage it slightly better … maybe the husband has not had to do so much of that during their lifespan and then, although it's difficult, seems to take it a lot better and although it's maybe unfamiliar tasks with cooking and things like that, they're very organised.’ (OT)

The way in which home care workers support family carers was explored, in response to the vignette. Home care workers can be a source of ‘strength’ for the family carer if they are struggling, and provide structure and support. They can be limited to how they support family carers, and are aware of providing advice but not dictating how nutritional care should be delivered.

‘He's going to get his strength from her, and maybe if he watched what she did with him, he might get an idea of how they can get in a routine or prompting her to eat her meals … Or maybe [he] just needs to be sat down and said look, we can do this, or get a food plan together … You're limited to what you can say. You can advise them but you can't tell them.’ (CW1)

It was important to the community dietitian that nutritional care was a shared responsibility. Although dietitians can be key in driving positive change towards improving someone living with dementia’s nutritional status, they are not solely responsible. Other health care professionals and family carers have a part to play in keeping someone well for as long as possible.

‘Also it's not just a dietitian's responsibility, I should say, in that everybody who's come into contact there; so the carers and any other health care professionals involved, can actually start to put in some steps to support them other than it just being the dietitian.’ (CD)

The concept of shared responsibility for nutritional care was explored further by another participant, who discussed the importance of family carers and people with dementia doing things ‘together’ that would contribute to improved nutritional care, such as shopping.

‘But they could make a shopping list; they can go shopping together. If she's putting loads of stuff in the basket, in her mind she's saying oh I like that, I like the thought of having that. So maybe having something that she likes, that she's going to eat, but as long as it's got the nutrition for her, I don't think it's that bad.’ (CW1)

### Theme four: improving nutritional care

Some participants explored how they felt nutritional care could be improved for people living with dementia at home. There was a recurring theme of the importance of increasing awareness of nutritional issues and more training in nutritional care for health care professionals, home care workers and family carers. For home care workers, a ‘generational knowledge gap’ regarding nutritional care is thought to have an impact for clients with dementia living at home. The perceived issues with meal delivery services were referenced by a few participants, but also the benefits that they can offer to both family carers and people with dementia.

Raising awareness of nutritional issues associated with dementia for patients and carers at point of diagnosis were recognised by one participant to be beneficial for the future. Family carers or the person with dementia may then notice nutritional issues before they escalate.

‘Step one is at the point of diagnosis and at that point we hope we've diagnosed them early enough where nutritional issues won't be a problem. But, if we raise awareness at that point, that could be helpful in the future.’ (GP)

The level of nutritional knowledge amongst participants varied. Aside from the community dietitian (‘the experts in the nutrition side of things’), participants felt that they lacked knowledge in the area of nutritional care for people living with dementia at home. Many felt that more training in this area would provide the knowledge required to identify nutritional issues and take appropriate action.

‘No. I've probably got no - zero knowledge of nutritional care in that sense … Are the microwave meals really that bad? I don't - we don't know. I don't know whether that - they're bad or not bad.’ (SW)

‘I think education for everybody because there's quite often lots and lots of people involved with a person … it would be just be a bonus to have it because we are aware, we realise that food and drink is really important and that it's an issue, but I wouldn't say that we actually have the right knowledge to maybe do what we need to do about it.’ (OT)

The age demographic of home care workers was considered an issue in the context of nutritional knowledge for one participant. Differences in school education over the years regarding cooking and nutrition was cited as the reason for this.

‘We have young members of staff coming in now that have absolutely no idea about nutrition and about - I think it's the schools. I think the schools need overhauling on it, I really do.’ (CW2)

The efficacy of meal-delivery services were raised by some participants. Most were dubious regarding their value, particularly with regards to the method of delivery. Participants felt that recipients of the meals were left confused and with little guidance regarding the meals, which inevitably resulted in them not being consumed.

‘ … all of their hot meals with big bold stickers on the front saying please eat immediately do not freeze. You have a look in their freezer and there's about 20 of these meals. The alarm bells start ringing.’ (CD)

‘They just turn up. You might have somebody by themselves or whatever and they turn up, they give them the meal, and then they leave, literally, at the door. That causes all sorts of problems.’ (SW)

Carers being able to spend time cooking with people living with dementia at home was suggested by participants. This would provide an enjoyable activity for the person with dementia, and increase the likelihood of meal consumption, therefore improving nutritional intake.

‘I'm quite sure that carers in the community that'll be commissioned would love to cook somebody a meal. I'm sure they would, and I'm sure that's a problem, and we would love them to cook the meal. But that's the - it's coming from the budget holders.’ (SW)

‘I just think that with dementia, to get their minds working as well and actively doing something, I think cooking - maybe someone could go in and cook twice a week with them and make meals up that can go in the freezer.’ (CW1)

### Reactions to the vignette

The vignette offered participants the opportunity to reflect on a scenario, based on a real-life situation (see Additional file 1). The vignette describes a husband’s experience whilst caring for his wife who has Alzheimer’s Disease. These include having to take over the cooking duties, concerns regarding eating adequately, weight loss, and the impact of memory loss. They were able to then relate their own experiences to the scenario, as well as how they felt about the situation. Participants recognised the scenario, and many accepted that it was something they see frequently in their professional practice.

‘It's kind of a familiar situation. It is something that we come across quite often.’ (OT)

‘Yeah, that is sad, but yeah, totally understand where he's coming from and I've seen that.’ (CN)

‘That's the kind of patient we see on a daily/weekly basis. Obviously really tough situation isn't it.’ (CD)

Participants sympathised with the family carer’s situation in the scenario, and were empathetic towards the change in his role, as well as how his wife’s dementia diagnosis may be affecting him.

‘In terms of her husband Peter, it's been a massive life change for him and his feelings and thoughts for the changing role; a quite devastating time for him really.’ (CD)

‘I think the bereavement, the grief, it must be so hard. If somebody dies and you have to come to some kind of acceptance at some point that you aren’t going to see that person again, but when that person is disappearing in front of you, I think that's so tough.’ (CW2)

As well as a focus on the health status of the person with dementia (‘there are red flags coming out around nutrition’ CD), the health of the family carer was also an area of concern for participants. This ranged from social and emotional support, to medical support.

‘Obviously he's not very aware of the whole situation, is he? He knows that his wife's got this dementia and he doesn't actually know how to deal with it because she's always been the actual role; she's looked after him. So now he just needs a little bit of backing up really doesn't he?’ (CW1)

‘Then, of course, we might want to look at him as well. What medication and support might he want?’ (GP)

Participants explored how home care workers could add value by offering practical support. This ranged from providing reassurance, to helping the family carer plan meals for the week.

‘So if they had someone come in and they taught them about what does she like to eat, how does she like to cook it, because it's guaranteed as soon as she gets in that kitchen she'll be saying oh I used to do it this way … Or maybe he just needs to be sat down and said look, we can do this, or get a food plan together.’ (CW1)

A range of solutions were offered by participants to help the couple. These included hot meal delivery to take the onus off the family carer, and modelling appropriate behaviours like eating meals together.

‘Depending on where they live … we can have hot meals delivered; quite a lot of villagers, like the local pub would deliver a hot meal and things like that. So I'd probably try to look in to what's available in their area. If he's concerned about whether he's cooking the right things and things like that, and also just to take a bit off of him, if he's not used to having to cook. Making sure that they've got the nutritious meals coming in would probably be something that I'd want to prioritise.’ (OT)

‘Sometimes if you sit down and eat with someone, they will eat as well. It's what they can see, not feel. She might be thinking oh I haven't eaten that so he's eaten, I'll eat. It's what you do … that makes a difference.’ (CW1)

## Discussion

The aim of this study was to understand the experiences and perceptions of health care professionals and home care workers who interact with people living with dementia at home relating to nutritional care. To the best of our knowledge, these findings contribute the first detailed interpretative phenomenological account of such experiences. The personal accounts highlight four superordinate themes central to this experience.

Health care professionals and home care workers feel that they have a responsibility towards the health of someone living at home with dementia. This was termed a ‘duty of care’ by some participants, who viewed it as an integral component of their professional role. This is important because health care, particularly nutritional care, often involves uncertainty and risk for individuals who are reliant on the competence of the health care professional [19]. Trust within health care relationships is thought to be reliant on health care professionals being non-judgemental listeners and ‘acting as a mirror for family strengths’ [20]. Surveys have found that older people in the UK feel that they are not involved in the wider home care system, which they find difficult to understand, and does not make the effort required to tailor care to their health needs [21]. Future service development must recognise that health systems are complex, and require integration of trust to enable successful outcomes [22].

It has been demonstrated that reductions in health and social care resource in England, UK are associated with increased mortality [23]. It is therefore important to ensure that all health care professionals and home care workers who visit people in their own homes, are able to take responsibility to identify potential health risks that may lead to deterioration of health, such as malnutrition. More focus is required on promoting holistic and non-siloed training and working approaches to care, particularly for those with complex long-term conditions [24].

Participants discussed the use of assessments and care plans, either conducting these themselves, or using them as a reference to ensure they understood the person living with dementia’s requirements. Care plans form an important part of a patient’s health journey, particularly in long-term conditions, and provide an opportunity for health services to measure outcomes. However, care plans that are focused on a chronic condition may not factor in the patient’s or professionals’ wider perspectives on goals or behaviour changes [25]. A trial of the effectiveness of a care plan delivered by memory clinics, and developed specifically for patients with Alzheimer’s Disease showed no difference in functional decline compared to usual care [26]. Care plans should have a broader focus, particularly as many people living with dementia also have other comorbid medical conditions [27]. Nutritional care should be an integral component of personalised care plans for people living with dementia at home, however more research is required concerning reducing the time burden of the care planning process for primary care practitioners and patients [28]. A potential remedy for this issue is to allow the patient and family carer to derive their own meaningful outcomes.

Participants felt that many issues regarding the effectiveness of nutritional care were as a result of limited time available for home care workers per visit. People with dementia may experience difficulties planning mealtimes, and may miss or leave meals if left unsupported [29]. Eating meals with others, playing background music, and allowing longer mealtimes may help, however more research is required to test such interventions, and whether home care workers could facilitate them [30]. Meal delivery was a solution proposed by some participants, however this was sometimes viewed as a sub-standard replacement to an extended home care visit. Delivered meals have been found to improve nutritional status and dietary intake in older adults (without a diagnosis of dementia) who reside in their own home [31, 32]. Despite this, some studies have found that this group tend to associate negative meanings with convenience food [33]. There is a paucity of studies exploring the effects of delivered meals for people living with dementia at home.

Participants were unanimous in their view that health care professionals and home care workers required more training and support regarding effective nutritional care. This view aligns with a recent survey that found that 86% of home care workers in the UK believe that dementia training would help them provide better care [34]. In the care home setting, studies have explored the importance of considering the meaning of the mealtime experience, and how residents can be empowered to make food and drink choices [35, 36]. More research is required into how similar approaches may be used within the ‘own-home’ setting, and with people living with dementia. Home care workers may be best placed to facilitate these approaches, however the complexities of their role and the training they require is under-researched [37].

### Strengths and limitations

This is the first study to report on the experiences and perceptions of health care professionals and home care workers regarding nutritional care for people living with dementia at home. Using a vignette during the semi-structured interview allowed participants to think creatively and provide responses that could be compared between professions. However, participants were asked to respond to the vignette from their own perspectives, which may have resulted in participants giving answers that where expected of their profession, rather than what they would do themselves. Participants were recruited through the lead researcher’s professional networks, which may have affected the interview dynamics and results. This was mitigated by ensuring transparency of the existing relationships between interviewer and interviewee, and by keeping a reflexive diary throughout the study. The diary allowed the lead researcher to actively explore how the information shared by participants impacted upon her own pre-existing beliefs and knowledge in order to understand the phenomenon of interest which was how health care professionals and home care workers provide nutritional care.

## Conclusion

This study explored the experiences of health care professionals and home care workers when providing nutritional care to people living with dementia at home. This group felt a sense of responsibility to ensure that people living with dementia received adequate care. The family caregiver was recognised by participants as needing support. However, challenges to providing effective nutritional care and support include limited time to spend with individuals, knowledge of appropriate food and drink choices, and decisions to replace carer support with meal delivery to reduce cost. The health care professionals and home care workers in this study recognised that working together as a team can improve care outcomes. Ideas for improvements focus on raising awareness of nutritional needs and developing training aids regarding nutritional care and dementia. The findings reinforce the importance of ensuring health care professionals and home care workers are provided with adequate training regarding identifying nutritional risks, helping family carers make appropriate food and drink choices, and preventing the risk of malnutrition in the dyad. Future research should focus on the efficacy of interventions to improve nutritional care from the health care professional and home care worker’s perspective, as ‘it’s what you do that makes a difference’.

## Additional files

Additional file 1: Vignette used during semi-structured interviews with health care professionals and home care workers (PDF 101 kb)

Additional file 2: Interview schedule used during semi-structured interviews with health care professionals and home care workers (PDF 190 kb)

## Data Availability

The datasets generated and analysed during the current study are not publicly available due to them containing personal and identifiable participant information.

## Abbreviations

CD: Community Dietitian
CLAHRC: Collaboration for Leadership in Applied Health Research and Care CN Community Nurse
COREQ: Consolidated Criteria for Reporting Qualitative research
CW: Care Worker
GP: General Practitioner
IPA: Interpretative Phenomenological Analysis
NIHR: National Institute for Health Research
OT: Occupational Therapist
SW: Social Worker

## References

[1] World Health Organisation: Dementia Factsheet. In*.*; 2017.

[2] Fauth EB, Gibbons A (2014). Which behavioral and psychological symptoms of dementia are the most problematic? Variability by prevalence, intensity, distress ratings, and associations with caregiver depressive symptoms. International Journal of Geriatric Psychiatry.

[3] Ikeda M, Brown J, Holland AJ, Fukuhara R, Hodges JR (2002). Changes in appetite, food preference, and eating habits in frontotemporal dementia and Alzheimer's disease. J Neurol Neurosurg Psychiatry.

[4] Kai K, Hashimoto M, Amano K, Tanaka H, Fukuhara R, Ikeda M (2015). Relationship between eating disturbance and dementia severity in patients with Alzheimer’s disease. PLoS One.

[5] van der Linde RM, Dening T, Matthews FE, Brayne C (2014). Grouping of behavioural and psychological symptoms of dementia. International Journal of Geriatric Psychiatry.

[6] Brodaty H, Donkin M (2009). Family caregivers of people with dementia. Dialogues Clin Neurosci.

[7] Alzheimer's Society: Challenges facing primary carers of people with dementia: Opportunities for research. In*.*; 2012.

[8] Mole L, Kent B, Hickson M, Abbott R: Family carers’ experiences of nutritional care for people living with dementia at home: An interpretative phenomenological analysis. *Dementia, forthcoming* 2019. 10.1177/1471301219872032 PMC7940801 31488020

[9] Skills for Care: Better domiciliary care for people with dementia: Best practice case studies from domiciliary care employers developing their workforces to support people with dementia. In*.* Leeds; 2014.

[10] Iliffe S, Jain P, Wong G, Lefford F, Warner A, Gupta S, Kingston A, Kennedy H (2009). Dementia diagnosis in primary care: thinking outside the educational box. Aging Health.

[11] Mole L, Kent B, Abbott R, Wood C, Hickson M: The nutritional care of people living with dementia at home: A scoping review. *Health Soc Care Community* 2018:n/a-n/a. 10.1111/hsc.12540 PMC6849562 29365369

[12] Schoenberg NE, Ravdal H (2000). Using vignettes in awareness and attitudinal research. Int J Soc Res Methodol.

[13] Tong A, Sainsbury P, Craig J (2007). Consolidated criteria for reporting qualitative research (COREQ): a 32-item checklist for interviews and focus groups. Int J Qual Health Care.

[14] Smith JA, Flowers P, Larkin M (2009). Interpretative phenomenological analysis : theory, method and research.

[15] Jarman M, Smith JA, Walsh SUE (1997). The psychological Battle for control: a qualitative study of health-care Professionals' understandings of the treatment of anorexia nervosa. J Community Appl Soc Psychol.

[16] Pietkiewicz I, Smith JA (2014). A practical guide to using interpretative phenomenological analysis in qualitative research psychology. Psychol J.

[17] QSR International: NVivo 11. In*.*, vol. Pro, 11.4.1.1064 (64-bit) edn; 2017.

[18] Hunt D, Smith JA (2004). The personal experience of carers of stroke survivors: an interpretative phenomenological analysis. Disabil Rehabil.

[19] Allinson M, Chaar B: How to build and maintain trust with patients. In: *The Pharmaceutical Journal.* vol. 297; 2016.

[20] Robinson Carole A. (2016). Trust, Health Care Relationships, and Chronic Illness. Global Qualitative Nursing Research.

[21] Sykes W, Groom C (2011). Older people’s experiences of home care in England. In.

[22] Gilson L (2003). Trust and the development of health care as a social institution. Soc Sci Med.

[23] Watkins J, Wulaningsih W, Da Zhou C, Marshall DC, Sylianteng GDC, Dela Rosa PG, Miguel VA, Raine R, King LP, Maruthappu M (2017). Effects of health and social care spending constraints on mortality in England: a time trend analysis. BMJ Open.

[24] Burger S-A, Hay H, Casanas i Comabella C, Poots A, Perris A (2018). Exploring education and training in relation to older people's health and social care. In.

[25] Burt J, Rick J, Blakeman T, Protheroe J, Roland M, Bower P (2014). Care plans and care planning in long term conditions: a conceptual model. Primary health care research and development.

[26] Nourhashemi F., Andrieu S., Gillette-Guyonnet S., Giraudeau B., Cantet C., Coley N., Vellas B. (2010). Effectiveness of a specific care plan in patients with Alzheimer's disease: cluster randomised trial (PLASA study). BMJ.

[27] Bunn Frances, Burn Anne-Marie, Goodman Claire, Robinson Louise, Rait Greta, Norton Sam, Bennett Holly, Poole Marie, Schoeman Johan, Brayne Carol (2016). Comorbidity and dementia: a mixed-method study on improving health care for people with dementia (CoDem). Health Services and Delivery Research.

[28] Edwards ST, Dorr DA, Landon BE (2017). Can personalized care planning improve primary care?. JAMA.

[29] Nygård L, Johansson M (2001). The experience and Management of Temporality in five cases of dementia. Scand J Occup Ther.

[30] Bunn DK, Abdelhamid A, Copley M, Cowap V, Dickinson A, Howe A, Killett A, Poland F, Potter JF, Richardson K (2016). Effectiveness of interventions to indirectly support food and drink intake in people with dementia: eating and drinking well IN dementiA (EDWINA) systematic review. BMC Geriatr.

[31] Wright L, Vance L, Sudduth C, Epps JB (2015). The impact of a home-delivered meal program on nutritional risk, dietary intake, food security, loneliness, and social well-being. Journal of Nutrition in Gerontology and Geriatrics.

[32] Zhu H, An R (2013). Impact of home-delivered meal programs on diet and nutrition among older adults: a review. Nutr Health.

[33] Peura-Kapanen L, Jallinoja P, Kaarakainen M (2017). Acceptability of convenience food among older people. SAGE Open.

[34] Carter D: Fix Dementia Care: Homecare. In*.*; 2016.

[35] Murphy JL, Holmes J, Brooks C (2017). Nutrition and dementia care: developing an evidence-based model for nutritional care in nursing homes. BMC Geriatr.

[36] Watkins R, Goodwin VA, Abbott RA, Hall A, Tarrant M (2017). Exploring residents’ experiences of mealtimes in care homes: a qualitative interview study. BMC Geriatr.

[37] D'Astous V, Abrams R, Vandrevala T, Samsi K, Manthorpe J: Gaps in understanding the experiences of homecare workers providing care for people with dementia up to the end of life: A systematic review. *Dementia (London, England)* 2017:1471301217699354. 10.1177/1471301217699354 28358269

