# Supplementary material for: ‘It’s what you do that makes a difference’ An interpretative phenomenological analysis of health care professionals and home care workers experiences of nutritional care for people living with dementia at home
Source: BMC Geriatr. 2019 Sep 10;19:250. doi: 10.1186/s12877-019-1270-4 (PMC6734271; doi:10.1186/s12877-019-1270-4)
Supplement: Supplementary file 1 — Vignette used during semi-structured interviews with health care professionals and home care workers (PDF 101 kb) [file 12877_2019_1270_MOESM1_ESM.pdf]

Vignette

My name is Peter, and Sandra and I have been married for 45 years. She is a retired nurse, was always good at looking after other people – including me! In fact, she has spent our married life looking after me. She used to do all of the shopping, cooking and cleaning until…well until a few years ago when things changed. The memory clinic said she had Alzheimer's disease which is a type of dementia as I understand it. Anyway, her memory got quite bad quite quickly and I have had to take over doing a lot of the things around the house. It hasn't been easy – I had never boiled an egg let alone plan a week of food shopping for two people and a cat (although the cat often eats the same as us as Sandra feeds it her food!).

Sandra does get quite confused – I often find packets of biscuits around the house in some strange places. She was such a good cook, and used to make everything from scratch. I don't cook so well, so I do rely on ready-meals for us both but I do worry whether we are eating the right things. I am still working a few days a week so we have a carer who comes in the mornings and she helps Sandra get up, washed and makes her breakfast and lunch. I worry whether Sandra eats enough, she leaves half of her meals when we eat together, and sometimes doesn't want to eat at all. The carer mentioned the other day that I need to get her some new clothes as her current ones are getting too big. I also worry whether she drinks enough; she forgets to drink cups of tea so I am forever heating them up in the microwave.

I don't like thinking of myself as Sandra's 'carer' as I am her husband. I feel like I have had to quickly change the way I react to things compared to what I used to be like. Back in the day, I wouldn't have had the patience for it, but now, if Sandra takes a whole pack of oven chips out of the freezer and puts them in fridge so that they defrost and have to be thrown away, I just think 'does it matter'? That is the main thing I have developed throughout this is patience. But you know the worst thing? Up until a few months ago, when Sandra was still able to make cups of tea, she asked me every time how I take my tea. After 45 years of marriage and now she can't remember how I take my tea – that really upset me.
