# Supplementary material for: ‘It’s what you do that makes a difference’ An interpretative phenomenological analysis of health care professionals and home care workers experiences of nutritional care for people living with dementia at home
Source: BMC Geriatr. 2019 Sep 10;19:250. doi: 10.1186/s12877-019-1270-4 (PMC6734271; doi:10.1186/s12877-019-1270-4)
Supplement: Supplementary file 2 — Interview schedule used during semi-structured interviews with health care professionals and home care workers (PDF 190 kb) [file 12877_2019_1270_MOESM2_ESM.pdf]

Interview Schedule

This semi-structured interview will consist of a series of open-ended questions. However, these questions will depend upon how much detail the participant wants to give. Due to the sensitive nature of this topic, participants will take some direction over this interview and some questions may be missed out or expanded upon. Minor amendments may be made as the interview progresses as issues may arise that the researcher had not considered.

<u>Introduction</u>

Thank you for taking part in this interview, I really do appreciate the time you have given. Before we begin, I want to make it clear that if you wish to skip any question(s) during the interview; or if you want to stop the interview, all you have to do is say; you don't need to give any explanation for doing so. I may also jot down a few notes if that is ok? I may also ask you to clarify or expand on things you say as we go through, this is to make sure I fully understand your experience.

Are you happy for us to begin?

<u>Baseline information</u>

Please can you confirm your profession?

How long have you been in this profession?

<u>Interview</u>

1. Can you tell me about your role, and how you come into contact with people living with dementia at home?

   Possible prompts: What do you do? What is involved as part of your initial/follow-up assessment?

2. What would you say are your main responsibilities regarding the care of people living with dementia at home?

   Possible prompts: What is involved? What support do you have from other professionals?

I am now going to read you a short case-study and will ask you some questions afterwards ….<read vignette>

3. Can you describe your feelings regarding Sandra and Peter's situation?

   Possible prompts: How did it make you feel? How do you feel it fits into your role? What could have been done differently?

4. Can you tell me about your experiences with working with family carers when it comes to nutritional care?

   Possible prompts: what happens? What is your involvement? What do you feel helps/hinders the family caregiver?

5. Can you tell me about how you think nutritional care could be improved for those living at home with dementia?

   Possible prompts: what might be the enablers/barriers to doing this? How would this affect your role? What impact do you think it would have on the person with dementia/the family caregiver?

6. **END** We have come to the end now, thank you for sharing your experiences with me today, is there anything else that you feel relevant to add that I may not have asked you?
